# Dermatology 2.0: Precision medicine for inflammatory skin diseases

**DOI:** 10.1111/jdv.70251

**Published:** 2025-12-26

**Authors:** Jeremy Di Domizio, Antoine Girardin, Curdin Conrad, Teofila Seremet, Michel Gilliet

**Affiliations:** ^1^ Department of Dermatology CHUV University Hospital and University of Lausanne Lausanne Switzerland

**Keywords:** bullous pemphigoid, chronic hand eczema, Darier's disease, digital platform, drug hypersensitivity reactions, immune modules, lichen planus, lupus, molecular profiling, mycosis fungoides, palmoplantar pustular psoriasis, precision medicine, psoriasisatopic dermatitis

## Abstract

Recent advances in immunology and transcriptomics have transformed dermatology, redefining inflammatory skin diseases as distinct molecular entities characterized by dominant immune pathways such as Th17, Th2, Th1, type I interferon (IFN) and myeloid modules. We transcriptionally delineated these immune modules, constructed a reference immune cartography to map patient skin biopsies and developed a digital platform that enables clinicians to analyse individual molecular profiles, facilitating precise diagnosis for clinically overlapping or diagnostically ambiguous dermatoses. Beyond diagnostic utility, this platform supports therapeutic decision‐making by aligning each patient's dominant immune module with the most appropriate targeted treatment. Analyses of treatment failures reveal that discordance between therapy and immune module dominance, as well as immune shifts between modules, account for most cases of non‐response. Realigning therapy with the patient's molecular profile restores efficacy and enables rational adaptation or repurposing of existing agents. By integrating molecular diagnostics with immune module‐guided therapy, this approach establishes a precision medicine framework that optimizes outcomes and advances personalized care for patients with inflammatory skin disease.

## Graphical abstract

Digital immune mapping of transcriptomics profiles from inflammatory skin disease biopsies enables precise molecular diagnosis, quantification of immune modules and personalized therapy selection by matching dominant immune signatures to targeted treatments.

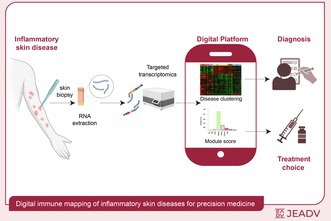

**Why was the study undertaken?**

- This study was undertaken to overcome the diagnostic uncertainty and therapeutic limitations of clinically heterogeneous and treatment‐refractory inflammatory skin diseases. We established a molecular platform integrating immune transcriptomic profiling of patient biopsies to enable accurate disease diagnosis and guide targeted therapy based on individual immune signatures.

**What does this study add?**

- This study introduces a digital transcriptomic platform that classifies cutaneous inflammation according to dominant immune modules (e.g., Th17, Th2 and IFN) and links each module to its corresponding targeted therapies. It demonstrates that treatment failures frequently arise from mismatches between therapeutic targets and immune dominance, which can be overcome by realigning therapy with the patient's molecular profile.

**What are the implications of this study for disease understanding and/or clinical care?**

- By integrating immune profiling with therapeutic decision‐making, this study establishes a precision medicine framework that improves diagnostic accuracy and optimizes treatment outcomes, thereby advancing personalized care for inflammatory skin diseases.

## WHY DO WE NEED PRECISION MEDICINE IN DERMATOLOGY?

Over the past 15 years, dermatology has undergone a true translational revolution. Advances in molecular technologies and immunology have revealed the key immune pathways that drive inflammatory skin diseases, and these discoveries have rapidly been translated into effective treatments with biologics and small molecules. The most relevant pathways are linked to T cell differentiation, including the Th17 axis characterized by IL‐23 and IL‐17A, the Th2 axis defined by IL‐4, IL‐5 and IL‐13, and the Th1 axis driven by IFN‐γ. Innate immunity also plays a central role, particularly through type I interferons (IFN) such as IFN‐α/β and through neutrophilic and IL‐1/IL‐36 pathways, with eosinophil‐driven inflammation forming another critical component.

Each of these pathways has been linked to specific skin diseases. Psoriasis is predominantly a Th17‐mediated disorder,^1^, ^2^ atopic dermatitis is driven by Th2 responses^3^ and lichen planus reflects Th1 activation.^4^ Lupus erythematosus is associated with type I IFN,^5^ while neutrophilic dermatoses such as Sweet syndrome, pyoderma gangrenosum and dissecans cellulitis show signatures of IL‐1 and neutrophilic genes.^6^ Wells syndrome, in turn, is defined by eosinophilic infiltration. The pathogenic importance of these immune axes is underscored by the remarkable efficacy of pathway‐specific therapies.

Despite these successes, clinical experience reveals that not all patients respond as expected. This discrepancy raises fundamental questions: is the diagnosis correct, are we targeting the right pathway, or could the immune profile have shifted under therapy? Precision medicine, which seeks to identify the dominant molecular pathway in each patient and align treatment accordingly, offers the best framework to resolve these uncertainties. Such an approach can shorten the patient's diagnostic journey, prevent therapeutic missteps and reduce costs associated with ineffective or unnecessary interventions.^7^

## IMMUNE MODULE DEFINITION AND CARTOGRAPHY DEVELOPMENT

Traditional efforts to define immune pathways have relied on the detection of effector cytokines. However, these molecules are often weakly expressed and insufficient to reliably distinguish between different inflammatory conditions. To address this limitation, we developed a transcriptomic strategy based on skin biopsies from well‐defined reference diseases that have well‐characterized responses to targeted therapies. By identifying genes that are differentially expressed in each reference disease relative to all others, we derived gene signatures (referred to as immune modules) corresponding to Th17, Th2, Th1, type I interferons and IFN‐stimulated genes (ISG), neutrophilic, macrophagic and eosinophilic pathways.^8^

Each module includes not only the canonical cytokines that define the pathway, but also upstream chemokines that recruit cytokine‐producing cells and downstream genes induced by these cytokines. Using an 80‐gene Nanostring panel, unsupervised clustering achieved perfect separation of reference disease samples based on their unique module expression. Independent test samples also clustered correctly with high sensitivity and specificity, outperforming clustering methods based on larger datasets of 600 immune genes or even 20,000 whole‐transcriptome genes.

This modular cartography enables systematic categorization of inflammatory skin diseases. Some disorders, such as psoriasis, atopic dermatitis, lupus erythematosus, lichen planus, neutrophilic dermatoses and Wells syndrome, are defined by a single dominant immune module. Others, however, are better characterized by distinctive combinations of modules. Bullous pemphigoid, for example, demonstrates co‐dominant Th2 and eosinophilic/macrophagic modules. Drug eruptions reveal a combination of dominant ISG, Th2 and macrophagic modules with variable Th1 activity. Morphea is characterized by dominant Th1 and myeloid signatures with a subdominant interferon signature. Thus, inflammatory skin diseases can be reliably distinguished either by the presence of a single dominant module or by unique combinations of existing modules, rather than by the discovery of entirely new immune pathways.

## CLINICAL APPLICATION FOR PRECISION MEDICINE

To translate this molecular cartography into clinical practice, we created a digital tool which allows clinicians to project Nanostring transcriptomic data from patient biopsies onto the established immune map. The platform generates visualizations as heatmaps or UMAP (Uniform Manifold Approximation and Projection) plots that cluster patient samples with reference disease cohorts based on similarities in immune gene expression profiles to facilitate diagnosis. In addition, it provides bar plots and quantitative readouts of the dominant immune module to guide treatment decision‐making. Cross‐centre validation has confirmed the reproducibility of results, and the platform is now shared with the global community via the Skin Science Foundation (https://skinsciencefoundation.org/).

**FIGURE 1:**
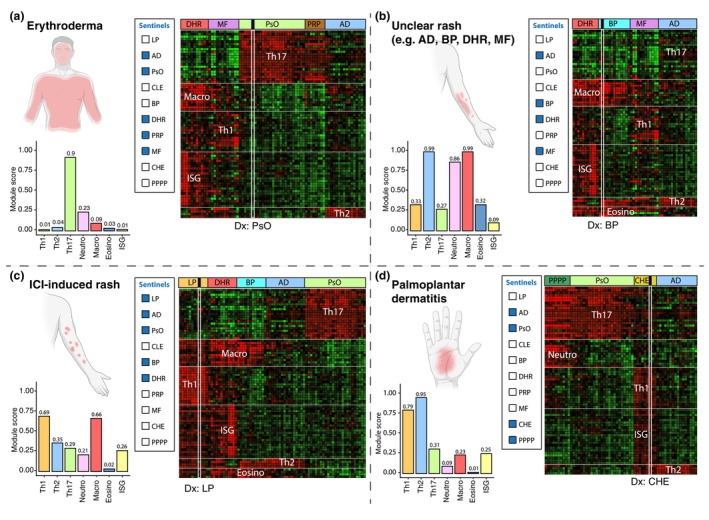
Our digital platform facilitates diagnosis of challenging cases. Analytical workflow of the digital platform for resolving challenging diagnostic cases using disease clustering and identification of dominant immune modules. (a) In erythroderma, our digital platform differentiates between AD, psoriasis, DHR, MF/Sezary syndrome and PRP by clustering transcriptomic profiles against a reference disease cartography. The erythroderma sample clusters unequivocally with psoriasis, displaying a dominant Th17 module, consistent with psoriatic erythroderma. (b) For generalized erythemato‐squamous eruptions with differential diagnoses of AD, prebullous pemphigoid, DHR, or MF, our digital platform identifies clustering with bullous pemphigoid, characterized by co‐dominant Th2 and macrophage‐associated modules. (c) Cutaneous manifestations induced by immune‐checkpoint inhibitors (ICIs) include psoriasis, lichenoid reactions, atopic eczema, bullous pemphigoid and DHR. Clustering of an ICI‐associated rash with the lichen planus reference group, showing a dominant Th1 module, confirms a lichenoid drug reaction associated with ICI therapy. (d) Palmoplantar dermatitis presents diagnostic challenges due to overlap among AD, chronic hand/foot eczema and non‐pustular or pustular psoriasis. Using predefined reference disease groups, our digital platform analysis reveals clustering within the CHE group, exhibiting co‐dominant Th1, Th2 and ISG signatures, confirming the diagnosis of chronic hand eczema.

The clinical utility of this molecular profiling approach is most evident in *diagnostically challenging* cases (Figure 1). Erythroderma, for instance, is notoriously difficult to classify on clinical and histopathological grounds alone, with differential diagnoses that include psoriasis, atopic dermatitis, drug eruptions, pityriasis rubra pilaris and Sézary syndrome. Digital mapping of erythroderma samples onto the immune cartography allows precise molecular classification that outperforms traditional diagnostic methods. Similarly, palmoplantar disorders are often difficult to distinguish clinically between pustular and non‐pustular psoriasis, atopic dermatitis and chronic hand or foot eczema. Module‐based clustering by the digital platform categorizes these palmoplantar diseases into four distinct molecular groups, enabling unambiguous diagnosis.^8^ In a cohort of 67 patients, clinical misclassification rates ranged from 29% to 63% (Seremet et al., 2025 in review), underscoring the urgent need to incorporate molecular diagnostics into routine management. The digital platform also provides clarity in common diagnostic dilemmas such as immune‐checkpoint‐inhibitor‐induced rashes, which may mimic psoriasis, lichenoid reactions, drug hypersensitivity eruptions, atopic dermatitis, or bullous pemphigoid. Beyond such complex cases, digital mapping can assist in resolving everyday differential diagnoses across the broader spectrum of inflammatory dermatoses included in its profiling database.

The immune cartography also serves as a valuable framework for *therapeutic decision‐making*. By quantifying the dominance of immune modules in pre‐treatment biopsies, clinicians can align each molecular profile with the most appropriate targeted therapy. In a retrospective study of 80 patients treated with either anti‐Th2 agents for atopic dermatitis or anti‐Th17 agents for psoriasis, seven patients were initially mismatched and failed to respond. Remarkably, all achieved clinical improvement after switching to the therapy corresponding to their dominant immune module. Similar observations in palmoplantar disorders further emphasize the need for molecular rather than purely clinical guidance in therapy selection (Seremet et al., 2025 in review).

**FIGURE 2:**
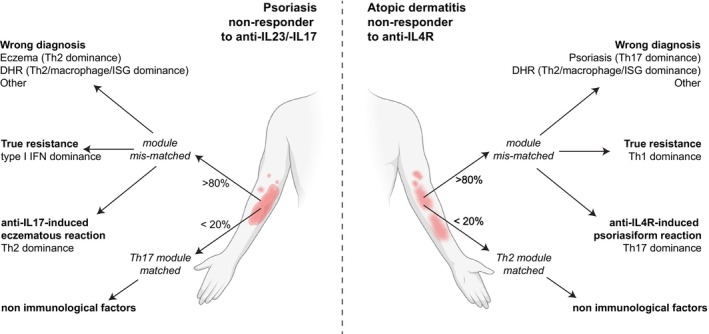
The digital platform clarifies the causes of treatment failures. Application of the digital platform enables identification of the immunologic mechanisms responsible for biologic therapy failure. In approximately 80% of treatment failures, the dominant immune module detected by our digital platform does not correspond to the pathway targeted by the administered therapy. Of these, 40% are attributable to misdiagnosis (e.g., Th2‐dominant eczema treated as psoriasis, Th17‐dominant psoriasis treated as eczema, or others), 30% reflect true biological resistance characterized by immune polarization shifts (a transition toward type I IFN dominance over Th17 in psoriasis or Th1 predominance surpassing Th2 in atopic dermatitis), and 10% represent paradoxical reactions associated with therapy‐induced immune axis and clinical phenotype switching (e.g., Th2→Th17 with psoriasiform lesions under anti‐IL‐4R or Th17→Th2 with eczematous lesions under anti‐IL‐17 treatment). In the remaining 20% of cases, the dominant immune module aligns with the therapeutic target, suggesting that resistance may result from pharmacologic nonresponsiveness.

A major advantage of molecular profiling is its ability to *clarify the causes of treatment failure*. The immune cartography provides direct evidence of the biological origin of non‐responses and thereby guides clinical management. In our cohort of 17 non‐responding cases, post‐treatment biopsies revealed a mismatch between the immune profile and the therapeutic target in 82% of patients (Figure 2). Among these, 41% were attributable to diagnostic error, most commonly eczema mistaken for psoriasis or vice versa.^8^

A further 30% represented true treatment resistance associated with immune shifts that explain loss of efficacy. In these cases, patients with atopic dermatitis resistant to IL‐4R blockade exhibited a shift from Th2 to Th1 dominance,^8^ whereas psoriasis resistant to IL‐17 or IL‐23 inhibitors developed type I interferon‐dominant profiles (Saidoune et al., 2025 in preparation).

Another 12% of non‐responses corresponded to paradoxical reactions, a distinct form of immune shift in which therapeutic inhibition of one pathway unleashes another. Paradoxical reactions are typically recognized clinically because they appear at new sites despite an initial therapeutic response,^9^ although their diagnosis can be challenging. For example, psoriasis patients treated with IL‐17 inhibitors may develop Th2‐driven eczematous eruptions,^10^ potentially reflecting the induction of TSLP expression by IL‐17 blockade,^11^ while IL‐4R blockade in atopic dermatitis can induce psoriasiform Th17 activation.^12^ Similarly, anti‐TNF therapy may precipitate paradoxical psoriasis characterized by interferon‐dominant signatures.^13^ Collectively, these findings support the concept of an immune shift, in which the transition to an alternative dominant module undermines therapeutic efficacy.

Only 17% of non‐responding lesions were matched to their intended therapeutic pathway, suggesting that resistance in these cases likely reflected additional, non‐immunological factors such as suboptimal drug dosing, pharmacokinetic variation, or non‐adherence. Recognizing the underlying mechanism of non‐response is therefore essential to guide rational therapeutic adaptation. In cases of diagnostic error, therapy should be redirected according to the true dominant immune profile. In cases of immune shift or paradoxical reactions, switching from a single‐pathway biologic to a broader‐spectrum agent that targets both the canonical disease signature and the emergent pathway (such as a JAK inhibitor targeting Th1, Th2 and Th17 signalling or TYK2 inhibitor principally targeting TH17 and type I IFN signalling) may be preferable.

**FIGURE 3:**
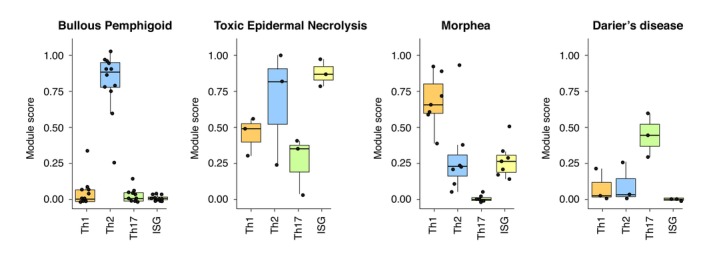
The digital platform reveals dominant immune modules for treatment repurposing. Boxplots illustrating expression of targetable immune modules in bullous pemphigoid (BP), toxic epidermal necrolysis (TEN), morphea and Darier's disease. Modules are considered dominant when their normalized expression exceeds 0.5 and falls within the top 30% of the most highly expressed modules. Bullous pemphigoid (BP) displays a dominant Th2 module, making it a suitable candidate for treatment with anti‐IL‐4R and anti‐IL‐13 blockers. In contrast, toxic epidermal necrolysis (TEN) exhibits co‐dominant Th1, Th2 and ISG modules, targetable by JAK1/2 inhibitors (inhibition of IFN‐γR, IL4R and IFNAR signalling). Morphea shows a dominant Th1 module inhibited by JAK1/2 inhibitors. Darier's disease shows a dominant Th17 module, supporting the rationale for using anti‐IL‐17 therapies.

Finally, module mapping expands opportunities for *therapeutic repurposing* (Figure 3). The identification of Th2 dominance in bullous pemphigoid provides a rationale for the successful use of IL‐4R inhibitors, as demonstrated in a recent multicenter phase 2/3 study^14^ (ClinicalTrials.gov identifier NCT04206553). The digital platform also reveals a dominant Th1/Th2 and ISG signature in toxic epidermal necrolysis, consistent with previous proteomic analyses and supporting potential responsiveness to JAK inhibitors.^15^ Similarly, morphea displays a predominant Th1 response, further supporting the rationale for JAK inhibition.^16^, ^17^ In contrast, Darier's disease exhibits a dominant Th17 module that could be therapeutically targeted with anti‐IL‐23 or anti‐IL‐17 agents, as previously reported.^18^ However, some studies have reported varying responses to Th17 targeting owing to potential compensatory inflammatory mechanisms.^19^ Thus, identification of the dominant immune module in individual patients using the digital platform may provide a powerful framework for selecting and repurposing the most appropriate targeted therapy.

## SUMMARY AND PERSPECTIVES

Dermatology is entering a new era in which the definition of disease is moving from morphology and histology toward molecular cartography. By classifying inflammatory skin disorders according to dominant immune modules or combinations thereof, clinicians can achieve more accurate diagnoses, improve therapeutic matching, monitor immune shifts and repurpose existing therapies for novel indications. The digital platform exemplifies how these insights can be integrated into practice, bringing precision medicine into the daily care of patients with complex inflammatory skin disease.

The next step will be to validate this approach through large, prospective multicenter studies, demonstrating that molecularly guided therapy selection achieves superior outcomes compared with conventional methods. This ‘Dermatology 2.0 paradigm’, defined by the integration of molecular profiling with clinical care, represents a fundamental conceptual shift that unites diagnosis, pathogenesis and therapy within a single precision medicine framework. This evolution promises to deliver truly personalized treatment strategies and to transform the management of inflammatory skin diseases in the years to come.

## AUTHOR CONTRIBUTIONS

Michel Gilliet: conceptualization and writing: original draft preparation. Jeremy Di Domizio: review, editing and figure preparation. Antoine Girardin and Teofila Seremet: review and editing.

## FUNDING INFORMATION

This work was supported by the Swiss National Science Foundation, Switzerland (310030B_182834, 310030_204835 and 4078P0_198470) to MG.

## CONFLICT OF INTEREST STATEMENT

Jeremy Di Domizio received a grant from Incyte and acted as a consultant for Signal26 Biotherapeutics. Antoine Girardin has no conflict of interest to declare. Curdin Conrad received grants from or acted as a speaker, consultant, expert, advisory board member for AbbVie, Amgen, Almirall, Boehringer Ingelheim, Bristol‐Myers Squibb, Celgene, Eli Lilly, Galderma, Incyte, Johnson & Johnson, LEO Pharma, Novartis, Pfizer, Samsung, Sanofi, Takeda and UCB. Teofila Seremet received grants from or acted as a speaker, advisory board member for Novartis, Sanofi, LEO Pharma, AbbVie, Lilly, Almirall, Pfizer, Galderma and MSD. Michel Gilliet received grants from or acted as a speaker, consultant, advisory board member for AbbVie, Almirall, Amgen, AstraZeneca, Beiersdorf, Eli Lilly, Galderma, Johnson & Johnson, Kyowa Kirin, L'Oréal, Louis Widmer, Merz Pharma, Novartis, Permamed, Pfizer, Pierre Fabre, Sanofi, UCB Pharma, Ultrasun, Verfora, Wellzia, Biogen, Merck, AVVA, BMS, Galderma, Innate Pharma, Owkin, Takeda.

## ETHICAL APPROVAL

Not applicable.

## ETHICS STATEMENT

Not applicable.

## Data Availability

Data sharing is not applicable to this article as no new data were created or analysed in this study.
